# Pathophysiological implications of hypoxia in human diseases

**DOI:** 10.1186/s12929-020-00658-7

**Published:** 2020-05-11

**Authors:** Pai-Sheng Chen, Wen-Tai Chiu, Pei-Ling Hsu, Shih-Chieh Lin, I-Chen Peng, Chia-Yih Wang, Shaw-Jenq Tsai

**Affiliations:** 1 Institute of Basic Medical Sciences, College of Medicine, National Cheng Kung University, 1 University Road, Tainan, 70101 Taiwan, Republic of China; 2 Department of Medical Laboratory Science and Biotechnology, College of Medicine, National Cheng Kung University, 1 University Road, Tainan, 70101 Taiwan, Republic of China; 3 Department of Biomedical Engineering, College of Engineering, National Cheng Kung University, 1 University Road, Tainan, 70101 Taiwan, Republic of China; 4 Department of Physiology, College of Medicine, National Cheng Kung University, 1 University Road, Tainan, 70101 Taiwan, Republic of China; 5 Department of Life Sciences, College of Bioscience and Biotechnology, National Cheng Kung University, 1 University Road, Tainan, 70101 Taiwan, Republic of China; 6 Department of Cell Biology and Anatomy, College of Medicine, National Cheng Kung University, 1 University Road, Tainan, 70101 Taiwan, Republic of China

**Keywords:** Cancer, Cardiomyopathy, Chronic kidney disease, Endometriosis, Metabolic diseases, Preeclampsia, Hypoxia

## Abstract

Oxygen is essentially required by most eukaryotic organisms as a scavenger to remove harmful electron and hydrogen ions or as a critical substrate to ensure the proper execution of enzymatic reactions. All nucleated cells can sense oxygen concentration and respond to reduced oxygen availability (hypoxia). When oxygen delivery is disrupted or reduced, the organisms will develop numerous adaptive mechanisms to facilitate cells survived in the hypoxic condition. Normally, such hypoxic response will cease when oxygen level is restored. However, the situation becomes complicated if hypoxic stress persists (chronic hypoxia) or cyclic normoxia-hypoxia phenomenon occurs (intermittent hypoxia). A series of chain reaction-like gene expression cascade, termed hypoxia-mediated gene regulatory network, will be initiated under such prolonged or intermittent hypoxic conditions and subsequently leads to alteration of cellular function and/or behaviors. As a result, irreversible processes occur that may cause physiological disorder or even pathological consequences. A growing body of evidence implicates that hypoxia plays critical roles in the pathogenesis of major causes of mortality including cancer, myocardial ischemia, metabolic diseases, and chronic heart and kidney diseases, and in reproductive diseases such as preeclampsia and endometriosis. This review article will summarize current understandings regarding the molecular mechanism of hypoxia in these common and important diseases.

## Introduction

In metazoan organisms, the oxygen delivery and cellular adaptation to oxygen deprivation are accelerated through the hypoxic signaling pathway in order to sustain oxygen homeostasis [130]. Lack of oxygen supply or an excessive oxygen consumption could result in insufficient oxygen levels for maintaining normal cellular function, a condition defined as hypoxia. Hypoxia may not be considered as the inequivalent to ambient oxygen concentration (21% oxygen) as many tissues physiologically function at levels equal to 5% oxygen or even as low as 1% oxygen [61]. Mostly, hypoxia is referred to the relatively low (generally < 2%) oxygen content compared to normal status in a given organ, tissue, or cell type. Hypoxia is a state of continuously lack of oxygen for a short (acute hypoxia, e.g., ischemia) or long (chronic hypoxia, e.g., chronic kidney disease, cancer) period of time. Therefore, a wider range of oxygen concentrations and feedback to acute stresses from seconds to days, even weeks to months, shall be put into consideration when referring the mechanisms of pathophysiological relevancies.

Hypoxia is usually considered to have pathological effects; however, it also involves in maintaining normal physiological functions. Taking human as an example, the central and peripheral chemoreceptors sense the reduction of oxygen tension and send signals to respiratory center in medulla and pons, where a series of processes are initiated to increase pulmonary ventilation and cardiac output to maintain normal functions of human body. Oxygen exchange occurs in the alveoli of lung with more than 95% of oxygen diffuses into the capillary vessels via the alveolar-capillary exchange system and then binds to hemoglobin. The oxygenated blood returns to left atrium through pulmonary vein. The heart then pumps out the oxygenated blood from left ventricle to its periphery to maintain the proper function of every single cell. In this minireview, we will not discuss the physiological effects of hypoxia but focus on pathological impacts of hypoxia in several key human diseases including cancer, cardiovascular diseases, chronic kidney diseases, metabolic diseases, preeclampsia, and endometriosis. Understanding the disease pathological process shall help us dissecting the molecular mechanisms of causing the disorders and designing better therapeutic regimens against them.

### Molecular basis of hypoxia in regulating gene expression

**Fig. 1:**
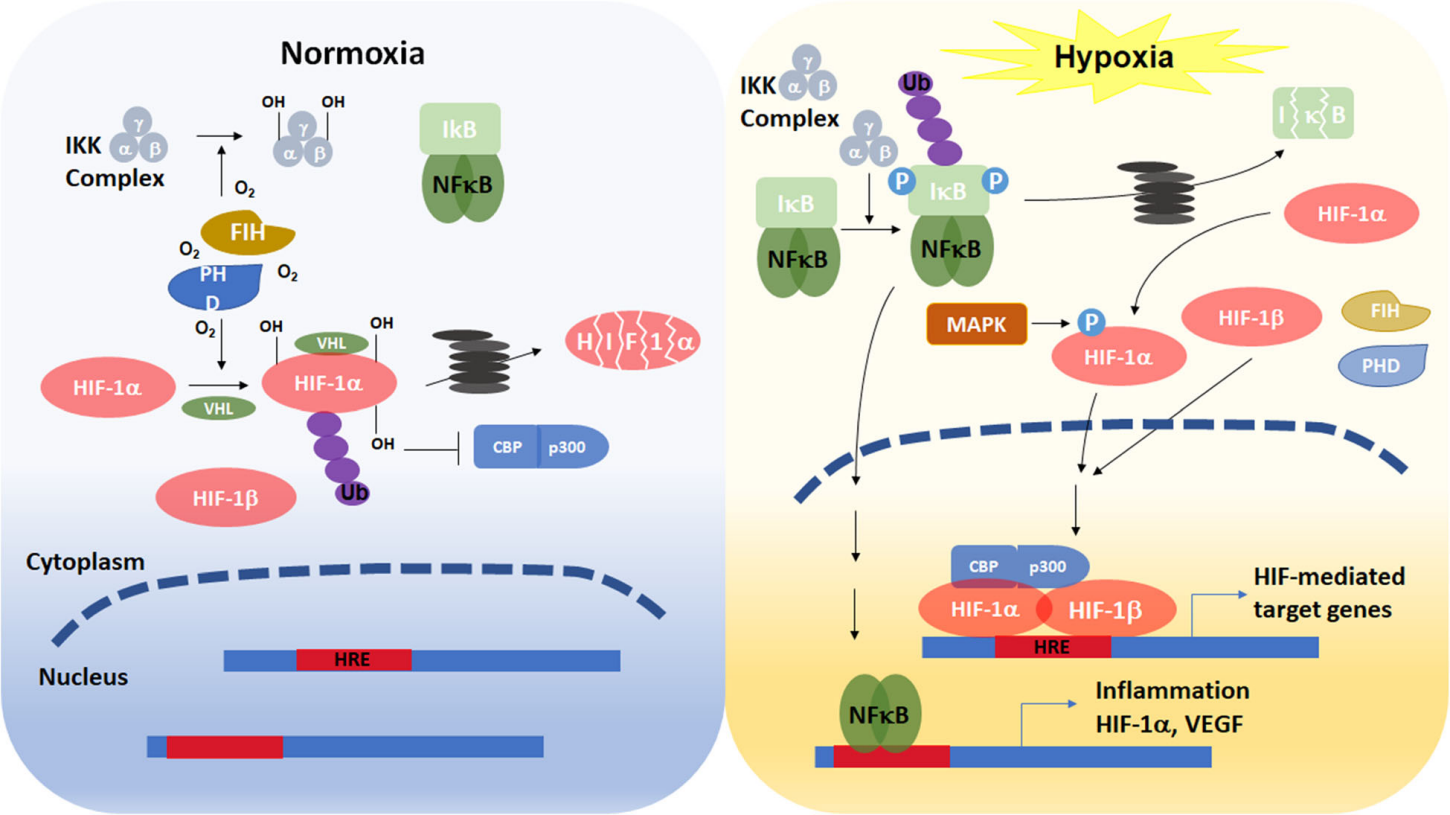
Schematic diagram illustrates the regulation of HIF-1α and NF-κB under normoxic and hypoxic conditions

Oxygen is essentially required by most eukaryotic organisms as a scavenger to remove harmful electron and hydrogen ions generated as by-products of mitochondrial oxidative phosphorylation. At the cellular level, adaptation involves a switch of energy metabolism from oxidative phosphorylation to anaerobic glycolysis, which increases glucose uptake, and expression of stress proteins related to cell survival or death [22]. All nucleated cells can sense oxygen concentration and respond to reduced oxygen availability in one of two distinctive ways. Alterations of preexisting proteins (such as phosphorylation or changing redox state) primarily occur in response to acute hypoxia (within minutes) while alterations in gene expression principally occur in response to chronic hypoxia (lasting from minutes to hours or longer). The expression of hypoxia responsive genes is mainly regulated by hypoxia inducible factor (HIF)- or nuclear factor-κB (NF-κB)- dependent manners. There are three HIF-αs (HIF-1α, −2α, and -3α) identified thus far [128]. All three HIFs dimerize with constitutively expressed HIF-1β (also known as aryl hydrocarbon nuclear translocator, ARNT) to form a heterodimeric functional unit. HIF-1α is expressed in most, if not all, human tissues [165] while HIF-2α and HIF-3α are expressed in more restricted tissues and developing stages such as fetal lung or developing vascular endothelium [39, 43, 146]. In reflecting to their specific tissue expression patterns, HIF-1α appears to play a general role in transcriptional regulation of all cells in response to hypoxia whereas HIF-2α and HIF-3α play more limited or specialized roles in oxygen homeostasis. Under normoxia, HIF-αs protein are hydroxylated by prolyl-hydroxylases (PHDs) and factor inhibiting HIF (FIH). These two oxygen-dependent enzymes are activated under normoxia and suppress HIF-αs activity via distinct mechanisms. PHDs catalyze the proline hydroxylation of HIF-αs so the E3 ubiquitin ligase, von Hippel–Lindau (VHL) can bind to HIF-αs protein and promote the degradation via the ubiquitin proteasome degradation pathway (Fig. 1). FIH hydroxylates the asparagine residue in the C-terminal transactivation domain of HIF-αs prevents the recruitment of transcriptional coactivator CREB-binding protein (CBP) and its homolog, p300, and thus inhibits HIF-αs transcription activity. In addition, PHDs and FIH can inactivate NF-κB by direct hydroxylation of inhibitor of κB kinase (IKK) complex [159]. Under hypoxia, insufficient oxygen inhibits the activity of PHDs, thus prevents HIF-αs from VHL-dependent protein degradation. In addition, mitogen-activated protein kinases (MAPKs) phosphorylate HIF-αs, which also increases the stability of α subunit. The phosphorylated HIF-αs protein translocate to the nucleus and associate with HIF-1β, which forms a HIF-1α/β heteroduplex and binds to the hypoxia responsive element (HRE) of target genes. Suppression of FIH activity under hypoxia results in increasing CBP/p300 recruitment to enhance the transcription of HIF target genes [60, 130]. Besides, lack of oxygen molecules prevents hydroxylation of IKK. Nonhydroxylated IKK complex promotes the phosphorylation, ubiquitination, and degradation of inhibitor of NF-κB (IκB). Therefore, NF-κB is released and translocates to the nucleus to regulate the transcription of target genes [32].

### Hypoxia and human diseases

#### Cancer

**Fig. 2:**
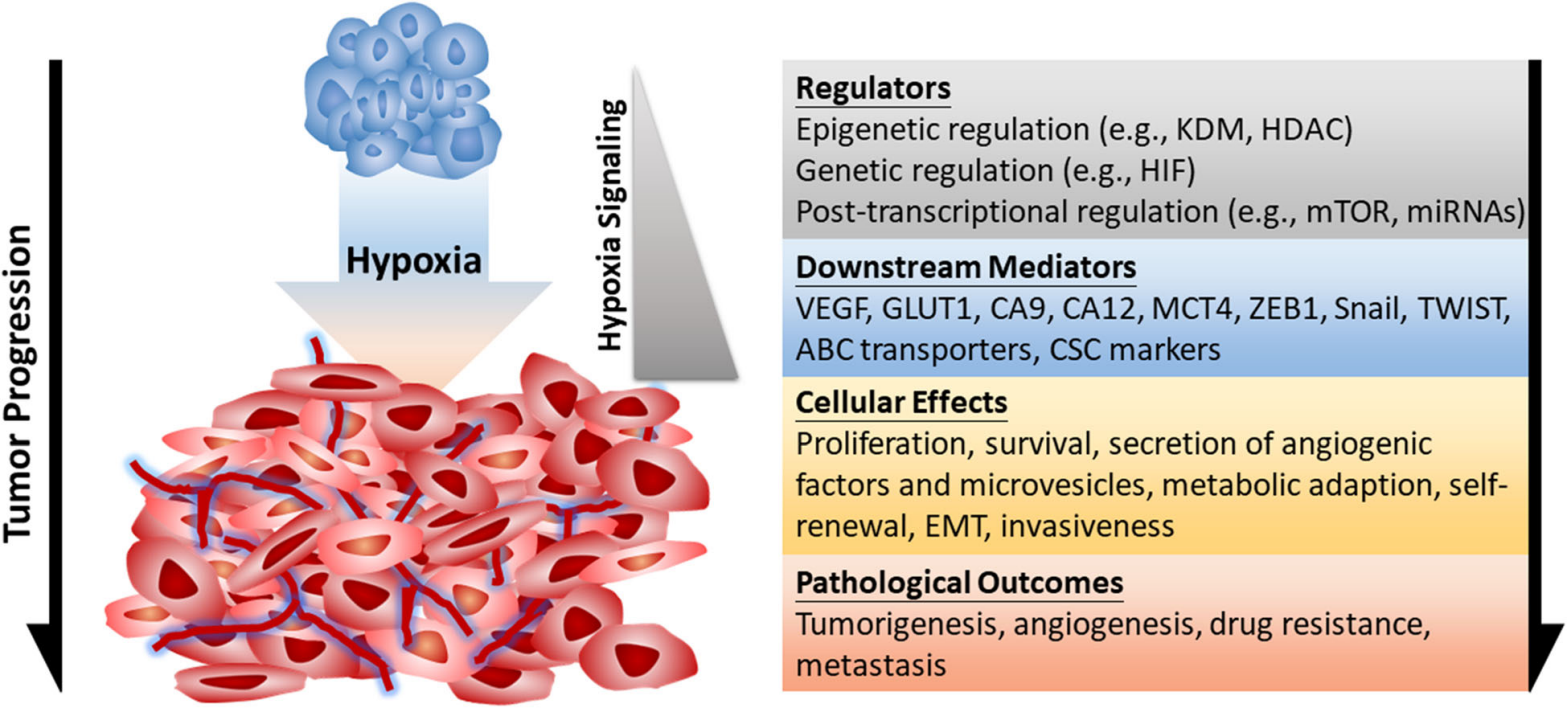
Hypoxia-regulated cancer progression. Hypoxia is a typical feature of tumor microenvironment, which contributes to initial tumorigenesis, induced angiogenesis, drug resistance, and cancer metastasis. The major upstream regulators (gray), functional downstream genes (blue), and resulting cellular consequences (yellow) under the control of hypoxia signaling are indicated

Impaired oxygen delivery and consumption are typical features of hypoxia in tumor microenvironment. The rise of hypoxia first comes from the restriction of oxygen diffusion in avascular primary tumors along with the higher oxygen consumption due to hyperproliferation of cancer cells [19]. Cellular responses to hypoxia are essential for tumor progression in many aspects, such as cancer cell survival, proliferation, epithelial-to-mesenchymal transition (EMT), invasion, angiogenesis, drug resistance, and metastasis [127]. According to findings involved in cancer biology from the initial tumor formation to advanced cancer dissemination, it is clear that tumor hypoxia is not only a hallmark of tumor microenvironment but also plays crucial roles in molecular and cellular responses to drive cancer progression. At the molecular level, hypoxia stabilizes HIFs to help cells adapting to hypoxic stress via transactivating downstream genes [130]. HIF-1α and HIF-2α are clinically correlated with advanced stages and poor survival of cancer patients [127]. In addition, the HIF signaling is essential for promoting the metastatic ability of cancer cells [117], suggesting that HIF pathway plays a vital role in cancer biology [127]. Furthermore, other pathways such as the mammalian target of rapamycin (mTOR) and the unfolded protein response (UPR) act cooperatively with HIF to regulate cellular functions [168]. Hypoxia suppresses mTORC1 activity through multiple pathways. Prolonged hypoxia causes energy stress that activates AMP-activated protein kinase (AMPK), which induces the transcription of regulated in development and DNA damage responses 1 (REDD1). REDD1 suppresses mTOR activity through tuberous sclerosis complex (TSC)1/TSC2-mediated mTOR inactivation [20]. Hypoxia can also inhibit mTORC1 activity through BCL-2 interacting protein 3 (BNIP3) and the promyelocytic leukemia (PML) tumor suppressor [168]. The suppressed mTORC1 activity results in decreased EIF4E-binding protein 1 (4E-BP1) dephosphorylation and thus sequesters eukaryotic translation initiation factor 4E (EIF4E) from cap-dependent translation initiation. The hypoxic inhibition of translation initiation is also reported to act through enhanced association with EIF4E-transporter (4E-T). Hypoxia induces endoplasmic reticulum (ER) stress sensors PKR-like ER kinase (PERK), inositol-requiring protein 1 (IRE1), and activating transcription factor 6 (ATF6) to induce the UPR pathway. The PERK-mediated phosphorylation of EIF2α results in globally suppressed translation initiation, while the other two ER stress sensors induce transcription through ATF6 and IRE1-activated X-box binding protein 1 (XBP1). These pathways have been long known to orchestrate a network with HIF to regulate gene expression at different molecular levels [168]. The widespread regulation by hypoxia/HIF signaling explains the molecular basis of hypoxia biology in cancer, from the stress (hypoxia), regulators (HIFs), targets (functional proteins), to phenotypes. Herein, we summarize hypoxia-regulated pathophysiological processes that play critical roles in cancer development and progression (Fig. 2).

##### Angiogenesis

The physiological diffusion of oxygen in microenvironment soon becomes a limiting factor during tumor growth, which stimulates new blood vessel formation (angiogenesis) to provide essential oxygen and nutrients for further tumor outgrowth. Tumor angiogenesis is tightly regulated by multiple pro- and anti-angiogenic factors. HIFs have been identified as master enhancers for vascular endothelial cell migration and proliferation. As a transcription factor, stabilized HIF-1α/β binds to HRE of target genes and transactivates their expression [48]. Among these genes, vascular endothelial growth factor (VEGF), platelet-derived growth factor B (PDGF-B), fibroblast growth factor (FGF), plasminogen activator inhibitor-1 (PAI-1), matrix metalloproteinases (MMP-2 and MMP-9), interleukin 8 (IL-8), and angiopoietins (ANG-1 and ANG − 2) are known as pro-angiogenic factors playing crucial roles during tumor angiogenesis [82, 94]. Moreover, VEGF and PAI-1 has also been found to enhance tumor angiogenesis under the control of HIF-2α [119, 138]. Among these secretory factors, VEGF, PDGF-B, FGF, ANG-1, and ANG-2 bind to their specific receptors VEGFR2, PDGFRβ, FGFR, TIE-1 and TIE-2, respectively, on vascular endothelial cells to activate cell proliferation, migration, tube formation, and vascularization [57, 113, 114], while other factors (e.g., MMPs, PAI-1) participate in remodeling extracellular matrix (ECM) and are also involved in local invasion of cancer cells [111].

##### Survival advantages from adaptive cellular responses

Malignant tumors tend to exhibit enhanced anaerobic glycolysis as their energy source. This metabolic shift enables cancer cells adapting to microenvironmental stresses including hypoxia [98]. Under hypoxia, cancer cell adapt to the reduced available oxygen and nutrient through upregulation of glucose transporters (GLUT1 and GLUT3), carbonic anhydrases (CA9 and CA12), pyruvate dehydrogenase kinases (PDK1 and PDK3), lactate dehydrogenase A (LDHA), phosphoglycerate kinase 1 (PGK-1), and hexokinases (HK1 and HK2) to cooperatively modulate a metabolic shift from oxidative phosphorylation to anaerobic glycolysis [92, 129]. Epigenetically, histone lysine demethylase 3A (KDM3A) removes demethylated histone 3 lysine 9 (H3K9me2) from PGK1 promoter to enhance HIF-1α-dependent PGK1 transcription thus facilitate glycolysis under hypoxia. Under hypoxia, the HIF-1α-dependent histone demethylase KDM4B induction removes H3K9me3 from the promoters of hypoxia-inducible genes involved in cell survival [124]. While the nonadaptive precancer/cancer cells undergo cell death, the hypoxia-induced anti-apoptosis pathways help other cancer cells survive. These evolutionally survived cells tend to express reduced pro-apoptotic factors such as Bcl-2-associated X protein (Bax), Bcl-2 associated agonist of cell death (Bad), and BH3 interacting domain death agonist (Bid), and enhanced anti-apoptotic factors such as B-cell lymphoma 2 (Bcl-2) and B-cell lymphoma-extra large (Bcl-xL) [50, 131]. Consequently, both of the poly (ADP-ribose) polymerase (PARP) cleavage and caspase activity are suppressed under hypoxia [40, 65]. These adaptive responses, including the metabolic and pro-survival shifts, not only cooperatively maintain the survival of cancer cell, but may also continually facilitate tumorigenesis from initial tumor formation to secondary tumorigenesis after/under treatment.

##### Epithelial-mesenchymal transition

Hypoxia/HIF signaling also regulates cellular behaviors, including migration/invasion, intra−/extra-vasation, colonization and tumorigenesis at distant organs, to drive cancer metastasis. EMT is a common process which enables noninvasive (epithelial-like) cancer cells to invade and metastasize (mesenchymal-like). It is now clear that hypoxia enhances the metastatic ability through promoting EMT of cancer cells. Hypoxia/HIF signaling directly facilitates the EMT gene profile to induce the invasiveness of cancer cells [117] through recognition of HREs of EMT transcription factors zinc finger E-box-binding homeobox 1 (ZEB1), snail family transcriptional repressor 1 (SNAI1), and twist family bHLH transcription factor 1 (TWIST1) genes [154]. Alternatively, HIF modulates Notch [123], transforming growth factor beta (TGF-β) [29], integrin-linked kinase (ILK) [26], tyrosine kinase receptors (TKRs) [34], Hedgehog [75, 140], AXL receptor tyrosine kinase [116], lysyl oxidase (LOX) [162], and protein 3-phosphoinositide-dependent protein kinase 1 [110] to indirectly facilitate EMT. In addition, histone deacetylase 3 (HDAC3) interacts with WD repeat domain 5 (WDR5) to enhance deacetylation of H3K4Ac in the promoters of EMT regulators under hypoxia [174]. These genetic regulations drive the phenotypic shift from epithelial-like (noninvasive) to mesenchymal-like (invasive), which help cancer cell motility during the multistep metastasis process, such as migration/invasion and intra−/extra-vasation.

##### Cancer stemness and drug resistance

Cancer stem cells (CSCs) expressing stem cell markers (e.g., CD133, CD44) and transcription factors (e.g., OCT3/4, SOX2, KLF4, c-MYC) have been identified to exhibit undifferentiated (stem cell-like) and tumorigenic properties [12]. Hypoxic microenvironment also facilitates the maintenance of CSCs [53]. It is known that both HIF-1α and HIF-2α induction activate OCT4, c-MYCc, SOX2 and enrich the expression of CD133- and CD44-positive cancer cells, along with the enhanced self-renewal functions and tumorigenic potential [4], while several studies proposed that cancer stemness is predominately controlled by HIF-2α [30, 62]. In addition to maintaining the unlimited primary tumor growth, CSCs have slow growing rate (quiescent) thus are also relatively insensitive to chemotherapy targeting proliferative cancer cells [25]. Thus, it is also suggested that CSCs within tumors determine the therapeutic efficacy and cancer prognosis, as the reservoir CSCs result in resistant subpopulation after chemotherapy and become the cellular sources for continued cancer propagation and recurrence. Irrespective of the involvement of CSCs in cancer recurrence, hypoxia signaling also activates several drug resistant pathways to protect cancer cells [151]. The nature of hypoxic region with poor vascularization may partly limit the diffusion of circulating drugs. Furthermore, HIF-1α induces the expression of multidrug resistance (MDR) genes under hypoxia. Expression of MDR gene products, the drug efflux pump protein ABC transporters, enables cancer cells to pump out intracellular chemotherapeutic drugs, thus reduces the therapeutic effects and enhances drug resistance [151].

##### Exosome secretion and priming

Exosomes released to microenvironment have been known to participate in intercellular communication since these extracellular microvesicles containing functional nucleic acids and proteins [100] . Recent studies demonstrated the clinical significance of hypoxic exosome in cancer. First, the contents of exosome, including proteins and nucleic acids, have significant changes under hypoxia [132] . Many of the proteins and RNAs enriched in hypoxic exosomes are canonical downstream gene products of hypoxia, such as HIF-1α, MMPs, and LOX [2, 69]. It is also known that the exosome secretion is enhanced in hypoxic cells to regulate cancer metastasis through the upregulation of small guanosine triphosphatase RAB22A [161]. Exosomes secreted by hypoxic cancer cells containing high levels of oncogenic proteins to enhance EMT, stemness and invasiveness through degradation of E-cadherin and activation of β-catenin pathway [115]. The currently identified extracellular secretion and functions of hypoxic exosomes make them as a possible hypoxic biomarker and therapeutic target.

##### Hypoxia regulates miRNA biogenesis

Current knowledge based on the large-scale genomic sequencing projects illustrated that the protein-coding transcriptional output is less than 2% in human genome. For recent 20 years, the roles of non-coding RNAs, such as miRNAs, have been extensively studied and their roles in cancer progression have been well-documented [134]. Here, we will discuss the regulation of hypoxia on miRNA biogenesis. The maturation of miRNA is a multistep process involving several protein factors. It is now established that both the global level of miRNAs and the expression of miRNA biogenesis factors (Dicer, Drosha, TARPB2, and DCGR8) are downregulated under hypoxia [10] . The cytoplasmic biogenesis factor Dicer is suppressed by HIF-1α-mediated proteasomal degradation or H3K27me3 demethylases KDM6A/B-dependent epigenetic modification [70, 156]. Several miRNAs including miR-103/107, let-7, and miR-630 were also reported to target and suppress Dicer expression [96, 122, 147]. Moreover, activation of EGFR pathway under hypoxia phosphorylates AGO2 and abolishes its interaction with Dicer, thus represses miRNA maturation and activity [135]. These pathways consequently result in either global or specific miRNA downregulation to promote cancer progression. Notably, many of the genes regulated by miRNAs are also canonical hypoxia/HIF signaling downstream genes, such as ZEB1, GLUT1, and VEGF, which further suggests that the hypoxia-regulated miRNAs act post-transcriptionally and synergistically with canonical hypoxia pathway of transcriptional regulation.

#### Cardiovascular diseases

Cardiovascular diseases are the leading cause of mortality worldwide, representing 1 of every 3 deaths in 2018. Hypoxia is one of the common features in the pathophysiology of a variety of cardiovascular disorders [1]. Heart failure with reduced left ventricular systolic function after myocardial infarction causes the insufficient oxygen in the body. Moreover, heart failure with preserved ejection fraction in patients eventually leads to systemic and pulmonary hypertension. Pulmonary hypertension also associates with other hypoxic pulmonary diseases, including chronic obstructive pulmonary disease and obstructive sleep apnea syndrome, and promotes inflammation and atherosclerosis [95]. **Atherosclerosis is** a chronic inflammatory disease that can increase the risk of myocardial infarction and stroke. The thickness of arterial wall causes hypoxia in the intima, reduces the perfusion of the tissue, and further stimulates proatherosclerotic processes, like inflammation, lipid synthesis, and angiogenesis [139].

##### Roles of NF-κB

As mentioned above, hypoxia simultaneously activates HIF and NF-κB signaling pathways to regulate numerous biological processes (Fig. 1). It should be noted that the crosstalk of HIF-1α and NF-κB plays an important role in ischemic cardiovascular disease. The hypoxia-induced HIF-1 upregulates NF-κB, which reciprocally activates the transcription of HIF-1α [38]. This forms a positive feedback loop to worsen the disease. In addition, NF-κB also induces many other target genes such as inflammatory cytokines. The inflammatory response leads to smooth muscle cell activation, resulting in neointima formation and occlusive plaque. Importantly, elevated serum inflammatory marker, such as IL-6 and tumor necrosis factor-α (TNF-α), in patients are correlated with their prognosis in hypoxic cardiovascular disease [76, 103].

##### Roles of Hif-1α

The functional consequences of HIF-1α in cardiomyopathy had been investigated using transaortic constriction (TAC) murine models to mimic pressure-overload heart failure human disease. A 3-week TAC in the mice with cardiomyocyte-specific deletion of *Hif1-α* fails to induce Vegf expression and neoangiogenesis. As a result, acute heart failure occurs due to the lack of enough oxygen being delivered to the rapidly increasing cardiac muscle cells [125]. Another study using *cardiomyocyte-* and endothelial cell-specific *Hif1-α knockout* mice showed 1-week TAC causes severe heart failure phenotype. Moreover, the myocardial capillary density is decreased in these mice, resulting from markedly increased endothelial cell apoptosis [164]. These results implicate the proangiogenic and cardio-protective effect of Hif-1α. Conversely, Krishnan and colleagues demonstrated that, after TAC, the *Hif1-α*^*+/−*^ mice have better cardiac function than wild-type mice. They also found Hif-1 promotes the expression of peroxisome proliferator-activated receptor γ (PPARγ), and activates lipid synthesis by engaging glycerolipid and fatty acid uptake genes, and, in turn, induces cell apoptosis [68]. These conflicting results suggest that Hif-1 mediates the complexity of adaptive responses. Hence, further research differentiating the roles of HIF-1 in cardiomyocyte is needed in order to acquire a clear picture.

##### Roles of miRNAs

In addition to the transcriptional regulation by HIF-1α and NF-κB, ischemic/hypoxia also modulates cardiofunction via miRNAs at the posttranscriptional level. Numerous miRNAs had been reported to be up- or down-regulated in patients with myocardial ischemia/reperfusion injury (Table 1). Among them, MiR-22, miR-134, miR-135a, miR-203, miR-144, miR-98, miR-18a, miR-210, miR-340-5p, miR-374a-5p, and miR-1192 exert protective effects in cardiovascular ischemic injury through downregulating their target genes [37, 41, 56, 77, 79, 112, 160, 163, 175, 183, 184]. On the other hand, a specific set of miRNAs *has been linked to cardiac dysfunctions in varied cardiac injury models.* For example, in intermittent hypoxia-induced myocardial damage, miR-146a-5p promotes cell death by targeting X-linked inhibitor of apoptosis protein [80] . MiR-327 reduces the expression of apoptosis repressor with caspase recruitment domain expression, and subsequently deteriorates myocardial ischemia/reperfusion injury [78]. MiR-429 accelerates ischemia/reperfusion injury by targeting mouse protein 25 and decreasing the protective effect of autophagy [189]. These data indicate that miRNAs do play important roles in regulating cardiovascular function after heart injury and imply they might be potential molecular targets for diagnosis or treatment of cardiovascular diseases. Indeed, miRNA-based therapies using modified oligonucleotides have been developed in treating different cardiovascular diseases. The critical role of miR-34a in cardiac ageing and function made it to be selected as a target for treating myocardial infarction [17, 144]. Targeting miR-34a by locked nucleic acid-modified anti-miR-34a attenuates adverse cardiac remodeling in myocardial infarction- or TAC-induced cardiac injury [15]. Similarly, anti-miR-92a and anti-miR-132 therapies are also effectively resistant to hypoxia-induced cardiac injury [13, 155]. Most recently, circulating extracellular vesicles-containing miRNAs attract great attention as promising biomarkers for early detection of cardiovascular diseases [3]. In lights of these recent advances, future studies focusing on elucidating key *miRNA diagnostic* biomarkers that can be targeted using mimetics or inhibitors to alleviate ischemic cardiovascular diseases are warranted.

**Table 1:** MicroRNAs involve in hypoxia-mediated human diseases

MicroRNA(s)	Change in expression	Regulatory gene(s)	Disease(s)
miR-18a	Up	BDNF	Myocardial infarction
miR-21	Up	PDCD4 Spry1	Heart failure Heart failure
miR-22 miR-34a	Up	SIRT1	Hypertrophy
miR-98	Down	DAPK1	Ischemia/reperfusion
miR-134	Up	NOS3	Ischemia/reperfusion
miR-135a miR-203	Down Down	PTP1B	Ischemia/reperfusion Myocardial infarction
miR-144	Down	FOXO1	Ischemia/reperfusion
miR-146a-5p	Up	XIAP	Intermittent hypoxia
miR-210	Up	EFNA3 PTP1B HIF-3α	Vascular remodeling
miR-327	Up	CRD	Ischemia/reperfusion
miR-340-5p	Down	Act1	Ischemia/reperfusion
miR-374a-5p	Down	MAPK6	Ischemia/reperfusion
miR-429	Down	MO25	Ischemia/reperfusion
miR-1192	Down	CASP3	Myocardial infarction

#### Metabolic diseases

##### Adipocytes and metabolic diseases

Adipose tissue is one of the least metabolically dynamic structure for lipid turnover and can also communicate with other tissues through secretion of adipocyte-derived hormones, growth factors, inflammatory cytokines, leptin, adiponectin, signaling lipids, fatty acids, and miRNAs packaged in exosomes [145, 188]. These adipocyte-secreted factors (adipokines) and fatty acids regulate systemic metabolism and play important roles in the development of metabolic diseases, such as metabolic syndrome, ischemic heart disease, stroke, obesity, type 2 diabetes mellitus, and cancer [7, 148]. White adipocytes in white adipose tissue, which is the most important lipid buffering organ, can either accumulate fatty acids in lipid droplets or supply fatty acids for other tissues determined by the balance between fatty acid synthesis (lipogenesis) and lipid breakdown (lipolysis and fatty acid β-oxidation) [63]. Conversely, brown adipocytes in brown adipose tissue and beige adipocytes in beige/brite adipose tissue more frequently produce heat through fatty acid β-oxidation in mitochondria (thermogenesis) [31]. Owing to their contributions to whole-body lipid homeostasis, both white and brown adipose tissues are considered primary targets for the treatment of obesity and type 2 diabetes mellitus [23, 28]. In addition, both white and brown adipose tissues are able to elaborate adipokines to control nutritional intake, sensitivity to insulin, and inflammatory processes in other tissues [28, 188]. Therefore, lipid metabolism and adipokines secretion in adipose tissue exert an impact on whole-body metabolism and are important for the progress of metabolic diseases.

##### Effects of hypoxia on adipocytes

Hypoxia is one of the mechanisms responsible for the development of metabolic changes and pro-inflammatory situations of white adipose tissue [150]. In obesity, due to the enlargement of adipocytes and increased distance from the vasculature, hypoxia occurs within the expanding white adipose tissue in ob/ob and dietary obese mice. Accordingly, white adipose tissues from obese people are subjected to intra-adipose tissue hypoxia and characterized by increased HIF-1α expression [67]. In vitro experiments show that HIF-1α and HIF-2α inhibit insulin signaling in both human and murine white adipocytes [120]. Moreover, chronic hypoxia has been suggested to be part of the pathogenic pathways leading to adipose tissue dysfunction [166, 178]. Hypoxia triggers reactive oxygen species (ROS) production, ER stress, inflammatory responses, angiogenesis, and adipocyte death [66, 150, 176, 185]. HIF also regulates the expression of various adipokine genes, such as increasing leptin, visfatin, apelin, TNF-α, *IL-1, IL-6*, VEGF, MMP2, MMP9, angiopoietin-like protein-4, macrophage migration inhibitory factor, and PAI-1 expression, while downregulating adiponectin and PPARγ expression in adipocytes [52, 150, 176].

Hypoxia alters several key metabolic processes including glucose uptake, glycolysis, oxidative metabolism, lipolysis, and lipogenesis in adipocytes. Hypoxia stimulates glucose uptake in adipocytes through HIF-1α-upregulated GLUT expression, and increases anaerobic glycolysis and lactate production by induction of glycolytic enzymes [121, 167]. Hypoxia also induces rearrangements of lipid metabolism in adipocytes. In response to hypoxia, extracellular fatty acid uptake is reduced by inhibition of fatty acid transporters (FATP1 and CD36) and transcription factors (PPARγ and C/EBPα) while lipolysis is increased in 3 T3-L1 adipocytes [179]. Another study shows that hypoxia inhibits lipogenesis by reducing PPARγ and fatty acid synthase (FAS), and induces basal lipolysis in visceral and subcutaneous human adipocytes [109]. In addition, hypoxia inhibits adipogenesis and differentiation in 3 T3-L1 adipocytes via HIF-1α-dependent upregulation of differentiated embryo-chondrocyte expressed gene 1 (DEC1/Stra13) and subsequent repression of PPARγ2 expression [182]. HIF-1α also suppresses expression of genes involved in fatty acid β-oxidation by repression of sirtuin 2-mediated deacetylation of PPARγ coactivator 1-α (PGC-1α) in white adipocytes [67]. Besides, studies indicate that obesity induces hypoxia in brown adipose tissue and causes the loss of its thermogenic capacity [149]. Other studies show that hypoxia is a trigger for brown adipose tissue whitening with diminished β-adrenergic signaling, enlarged lipid droplets, and loss of mitochondria in the cells [137]. Increased HIF-1α and suppressed uncoupling protein 1 (UCP1) expression with lower fatty acid β-oxidation are observed in hypoxic brown adipose tissue [136]. Therefore, hypoxia alters lipid metabolism in adipocytes mainly by inhibiting lipogenesis and decreasing fatty acid β-oxidation.

##### Effects of hypoxia on other cell types

The effects of hypoxia on lipid metabolism are also studied in other cell types related to metabolic diseases. As fatty acid β-oxidation takes place inside mitochondria and requires oxygen, fatty acid metabolism has to be modified other than energy production under hypoxia. Furthermore, the major source of cytoplasmic acetyl-CoA from glucose converted citrate is prohibited under hypoxia due to the inhibition of the TCA cycle, so alternative sources of fatty acid precursors have to be exploited. Uptake of extracellular fatty acids for triacylglycerol synthesis is promoted by HIF-1α-induced PPARγ in cardiomyocytes under hypoxia [68]. Extracellular fatty acid influx and lipid droplet accumulation are enhanced via HIF-1α-mediated induction of fatty acid binding protein 3 and 7 (FABP3 and FABP7), while de novo lipogenesis is repressed in glioblastoma and breast cancer cells under hypoxia [14]. To maintain certain level of lipogenesis under hypoxia, production of fatty acid precursors, citrate and acetyl-CoA, are supported through reductive glutamine metabolism in several cancer cells [46, 105] and brown adipocytes [180]. HIF-induced isocitrate dehydrogenase 1 and 2 (IDH1 and IDH2) contribute to the preservation of citrate levels via conversion of α-ketoglutarate to isocitrate and its subsequent reductive carboxylation to produce citrate from glutamine under hypoxia [101]. Adequate fatty acid synthesis is further supported by HIF-1-dependent activation of sterol regulatory element-binding protein 1 (SREBP1), which in turn upregulates the expression of FAS in breast cancer cells [45]. Hypoxia also induces lipid droplet accumulation by upregulating two enzymes of the triacylglycerol biosynthesis pathway, acylglycerol-3-phosphate acyltransferase 2 (AGPAT2) and lipin-1 in different types of cancer cells [106, 152]. Furthermore, hypoxia-induced lipid droplet accumulation is accompanied by the inhibition of fatty acid β-oxidation though HIF-1 and HIF-2-dependent downregulation of PGC-1α and carnitine palmitoyltransferase 1A (CPT1A) in both hepatoma and renal cell carcinoma cells [35, 90]. Therefore, hypoxia may result in different adaptation of lipid metabolism depending on the cell types.

##### Effects of hypoxia on animal models of obesity

Since hypoxia is shown to inhibit fatty acid β-oxidation as a promoter of obesity and inhibit lipogenesis as a suppressor of obesity, several animal studies based on the overexpression or inhibition of HIFs in adipocytes suggest that HIF activation either promotes or inhibits metabolic diseases. As a promoter of obesity, mice overexpressing Hif-1α in adipocytes have elevated obesity and insulin resistance associated with increased inflammation and fibrosis [49, 59]. Adipocyte specific *Hif-1α* or *Hif-1β* knockout, or inhibition of Hif-1α by inhibitors decrease obesity and insulin resistance in mice fed with high-fat diet [58, 74, 142]. In agreement, adipocyte-specific *Phd2* ablation enhances adiposity in mice under normal chow diet (low-fat) with lower expression of adipose triglyceride lipase (ATGL) and suppresses lipolysis in white adipocytes [102]. These obesity-promoting effects can be correlated with the capacity of Hif-1 to downregulate fatty acid β-oxidation in white and brown adipose tissue [67, 136]. On the other hand, a number of studies have shown that Hif activation decreases obesity and Hif inhibition increases obesity indicating hypoxia as a suppressor of obesity. Accordingly, transgenic mice overexpressing an adipose tissue-specific dominant negative *Hif-1α* mutant developed severe obesity, insulin resistance, and accumulated enlarged lipid droplets in brown adipose tissue with decreased mitochondrial biogenesis after high-fat diet [187]. Another group shows that transgenic mice with adipose tissue-specific knockout of *Phd2* (for constitutive expression of Hif) are resistant to high-fat diet-induced obesity with fewer lipid droplets in white adipose tissue and increased Ucp1 expression in brown adipose tissue [97], while adipocyte-specific knockout of *Phd2* induces adiposity in mice under chow diet (low-fat) [102]. Similarly, phenotypes are also observed in mice globally lacking *Fih*, which are resistant to high-fat diet-induced weight gain and hepatic steatosis [186]. Taken together, these studies suggest that Hif-1α may stimulate the thermogenic functions of brown adipose tissue to conquer high-fat diet-induced obesity, which is contradictory with other findings that Hif-1 suppressed expression of genes involved in fatty acid β-oxidation in white and brown adipose tissue leading to obesity [67, 136].

**Fig. 3:**
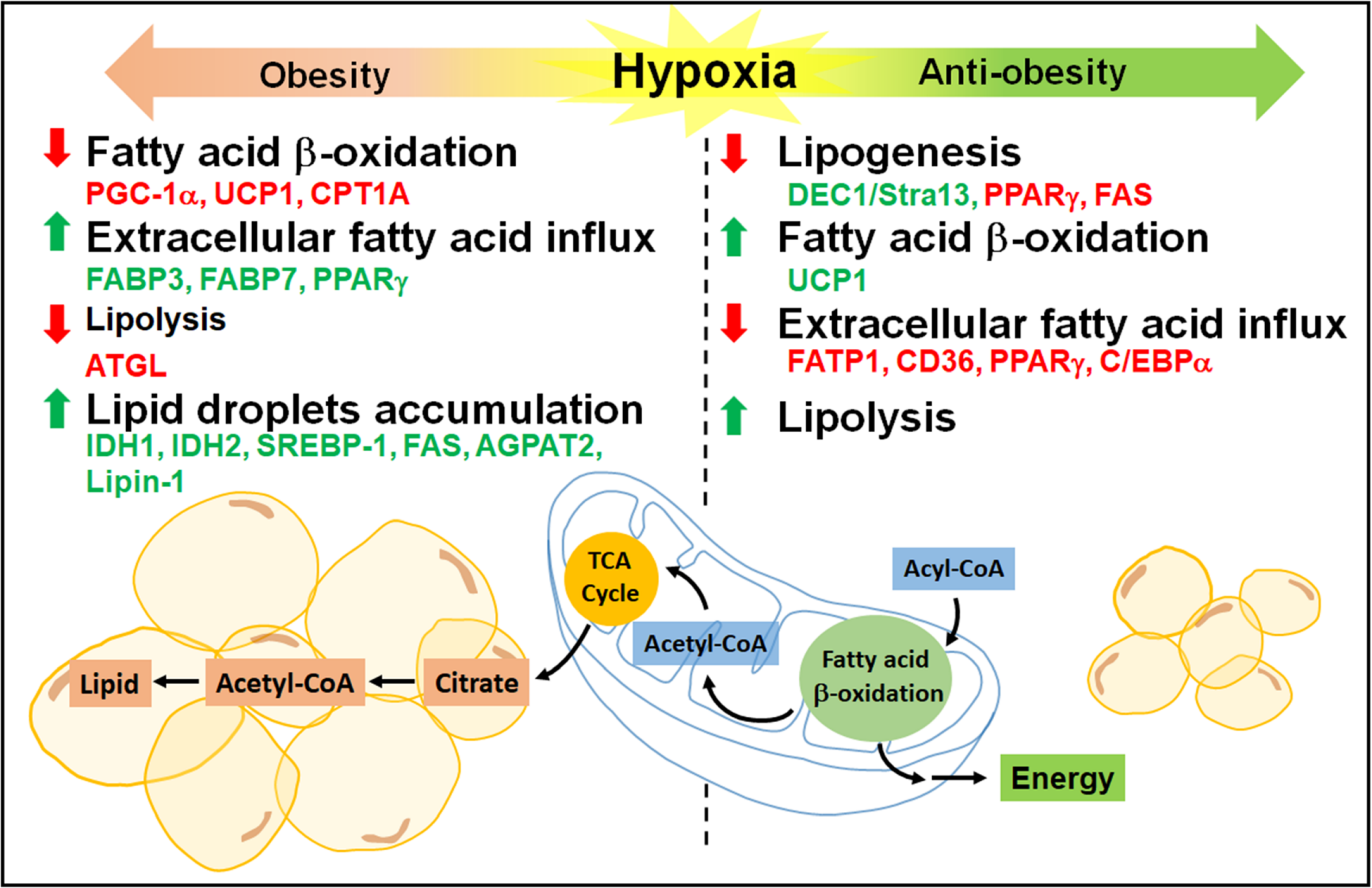
Hypoxia-regulated lipid metabolism related to obesity. Hypoxia is shown as a promoter or a suppressor of obesity by regulating lipid metabolism. The major changes (increases shown in red; decreases shown in green) involved in fatty acid β-oxidation, extracellular fatty acid influx, lipolysis, lipogenesis and lipid droplet accumulation under hypoxia for its promoting obesity or anti-obesity effects are summarized

In summary, studies investigating the functions of HIFs in adipocytes and other metabolic diseases revealed the conflicting results due to different experimental conditions (Fig. 3). The roles of HIFs in these studies were discovered primarily through the analysis of conditional *Hif*-knockout mice or through some pharmacological HIF inhibitors. These inconsistent consequences might be also due to the complexity of metabolic regulation with the complicated roles of HIFs that extend further than lipid metabolism. Therefore, the actual functions of HIFs in adipocytes and related metabolic diseases must be carefully interpreted relative to different physiological conditions.

#### Kidney diseases

Acute kidney injury (AKI) is notorious for its correlation with the development of chronic kidney disease (CKD). AKI is a rapid failure of kidney function, usually caused by decreased blood flow, toxic exposure, and ureteral obstruction. As the injuries are removed, renal cells undergo repair process. However, some of the AKI patients fail to fully recover. Instead, CKD starts to develop, which is characterized by the progressive loss of the kidney function [27]. Events include tubular atrophy, vascular rarefaction, and hypoxia, which lead to renal fibrosis and functional loss of kidney. Ultimately, CKD will develop into the end-stage renal disease (ESRD). Today, the only treatments for ESRD are dialysis and kidney transplantation [91]. Kidney fibrosis, including glomerulosclerosis and tubulointerstitial fibrosis, a phenomenon of excessive ECM deposit and accumulation, has been recognized as the hallmark of CKD and the major pathway leading to ESRD [107]. Fibrosis is the terminal pathway involved in the continuous progression of CKD and it is a consequence of failed recovery after kidney damage. When injuries happen, cells in kidney including fibroblasts, tubular epithelial cells, endothelial cells, pericytes, lymphocytes, and macrophages, undergo wound healing programming in an attempt to repair tissues. Sometimes, the damage is too severe for cells to overcome. Subsequently, they become victims and even act to fuel the fibrogenesis progressively [18]. As the diverse origin of cells involved in kidney fibrosis, it is complicated to figure out a comprehensive therapy for treating CKD patients. Today, there is still no effective ways to prevent CKD from getting worse. Several publications have pointed out that hinder renal fibrosis from getting worse is a potential way to prevent AKI-CKD transition and delay ESRD development [11, 16].

##### Hypoxia in kidney fibrosis

**Fig. 4:**
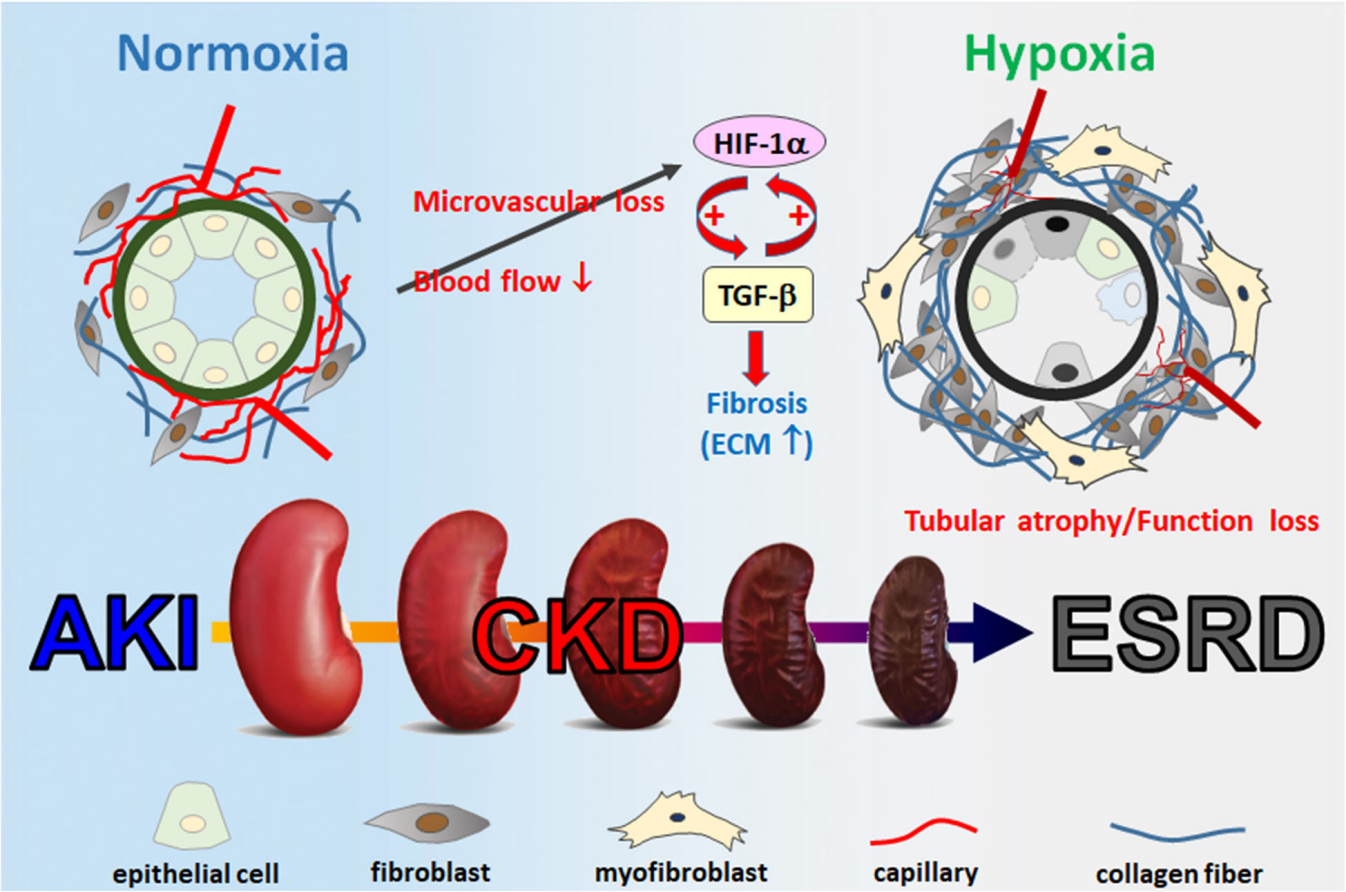
Schematic diagram of the hypoxic process linked to kidney diseases. During the development of CKD, vascular endothelial cells died and causing atrophy of microvessels. This will cause local hypoxia between tissues and cause inflammatory reactions. Hypoxia induces the expression of TGF-β, which leads to fibroblast transformation into myofibroblast, increases ECM production, and causes fibrosis. Renal tubular epithelial cells may encounter cell cycle arrest, apoptosis, autophagy, and finally shrink during renal fibrosis

Tubular injury may lead to renal microvascular loss, which restricts downstream blood flow from glomerular capillaries and contributes to the development of renal hypoxia. Loss of microvasculature, reduced oxygen dispersion, and metabolic abnormality of cells in the kidney are the main causes of the hypoxic state. The initiation of hypoxia is one of the main causes of AKI, which can increase levels of HIF-1α, followed by the induction of TGF-β signaling. During the process of kidney fibrosis, hypoxia and TGF-β1 signaling are excessively upregulated. AKI can be gradually developed into CKD if TGF-β signaling remains hyperactivated for a period of time. Thus, hyperactivation of TGF-β signaling is responsible for the renal fibrosis and can also be identified as the hallmark of CKD. Hypoxia is common at the beginning of kidney fibrosis, which promotes HIF-1α expression that contributes to both a result and a cause of renal fibrosis. Hyperactivation of myofibroblasts is responsible for kidney fibrosis as they continuously produce collagens, fibronectin, and vimentin, which contribute to tubulointerstitial fibrosis and local tissue hypoxia [72, 143]. Prolonged hypoxia further promotes renal fibrosis by increasing the synthesis of type I and type IV collagen and inhibiting the expression of MMP-1 in human renal fibroblasts [108]. By using kidney-specific *Vhl*^−/−^ mice, which have a stable expression of Hif-1α in kidney, researchers found that these transgenic mice exhibit more severe interstitial fibrosis after conduct 5/6 nephrectomy [64]. In contrast, intraperitoneal injection of HIF-1α inhibitor YC-1 protects unilateral ureter obstruction mice from kidney fibrosis development [126]. These data indicate that HIF-1α has a pivotal role in mediating kidney fibrosis (Fig. 4). However, the underlying mechanisms by which HIF-1 accelerates kidney fibrosis remain unclear.

#### Preeclampsia

Preeclampsia is a severe gestational complication featured by new onset of high blood pressure after 20 weeks of gestation along with signs of proteinuria, abnormally high serum creatinine, or damaged liver function [36]. Its complications remain a major cause for morbidity and mortality in pregnant women and fetuses. Defective trophoblast invasion into the decidualized endometrium leads to poor transformation of uterine spiral arteries from high to low resistant vessels [42]. This deficient vessel remodeling causes a sustained hypoxic environment implicating the development of preeclampsia. Thus, placental ischemia and hypoxia is a major cause of preeclampsia.

##### Primary cilium and preeclampsia

**Fig. 5:**
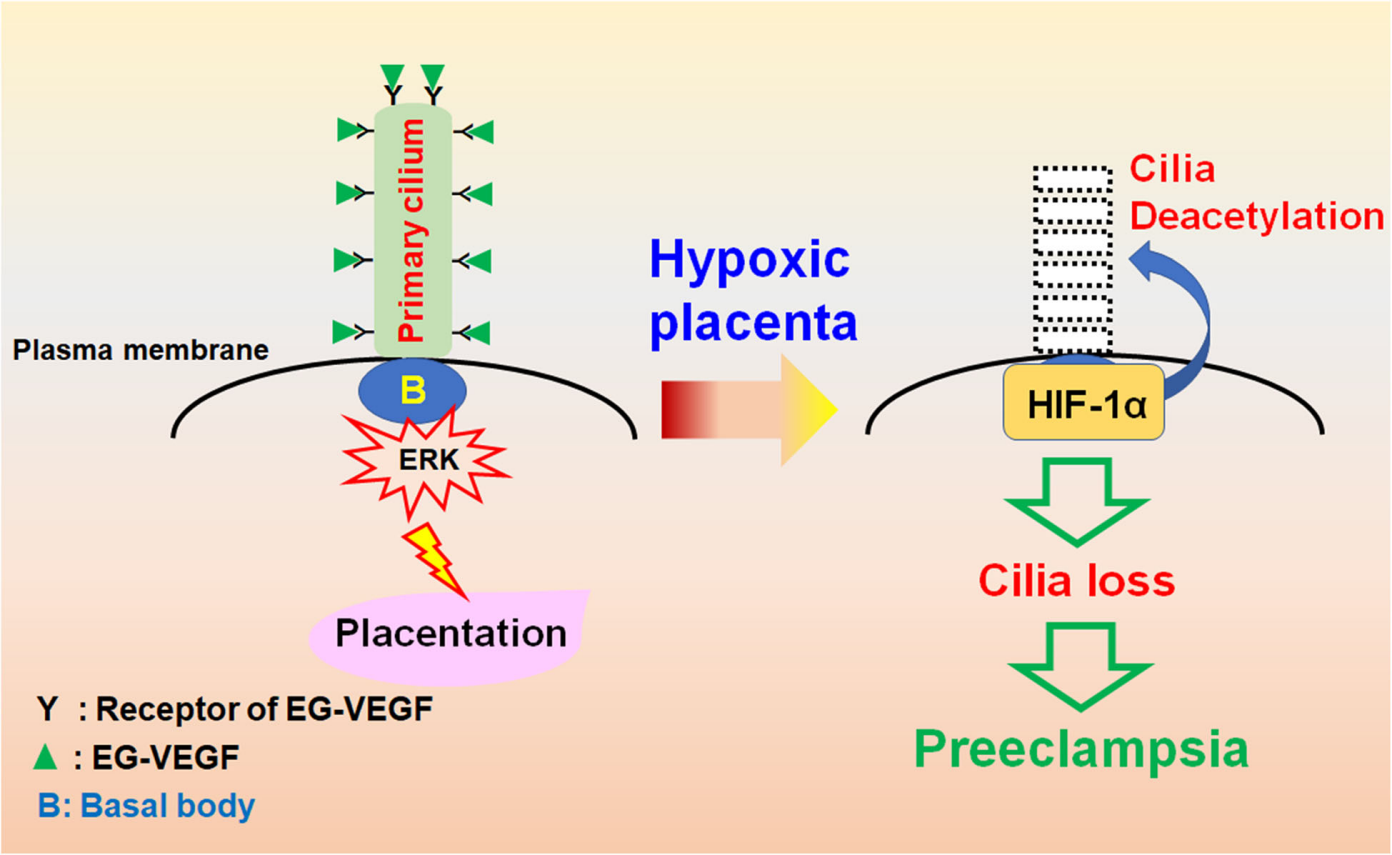
Potential role of hypoxia in developing preeclampsia. Binding of EG-VEGF to its receptor on the primary cilium activates ERK signaling at the basal body for proper placentation. Under hypoxic condition, however, HIF-1α translocates to the base of cilia and induces cilia deacetylation, thus leading to ciliary resorption. The hypoxia-induced ciliary defects contribute to the development of preeclampsia

Defective trophoblast invasion leads to placental hypoxia. The trophoblast invasion is regulated by endocrine gland-derived vascular endothelial growth factor (EG-VEGF, also known as prokineticin 1) [51]. EG-VEGF induces the expression of MMPs by triggering ERK signaling cascade for proper trophoblast invasion [157]. The receptor of EG-VEGF localizes to the primary cilium, a cellular protrusion atop from the centrosome (Fig. 5). When EG-VEGF binds to its receptor, ERK signaling is initiated from the base of the primary cilium, and then transduced throughout the cytoplasm. Inhibition of EG-VEGF signaling or disruption of primary cilia alleviates trophoblast invasion in vitro [158]. More importantly, these phenotypes are also observed in pregnant women who suffered from preeclampsia. These data suggest the important roles of EG-VEGF and primary cilia in preventing placental hypoxia.

Primary cilium functions as a sensory hub for transducing environmental chemo- and/or mechano-signaling into the cells for proper development and differentiation. Growing body of evidence supports the important role of primary cilium in maintaining embryo development. Interestingly, hypoxia suppresses primary cilium formation [71], and elevated levels of HIF-1α in the human trophoblasts have been linked to the development of preeclampsia [5], suggesting placental hypoxia-activated HIF-1α plays a key role in the pathogenesis of preeclampsia. It has been shown that HIF-1α translocates to the base of primary cilium for the resorption of primary cilia in the nutrient deprivation model, suggesting its non-genomic function in regulating primary cilium formation (Fig. 5).

##### HIF-1α in ciliogenesis

Despite the role of hypoxia in preeclampsia has been demonstrated for long, the underlying molecular mechanism remains unclear. Trophoblast invasion is triggered by the binding of EG-VEGF to its congenital receptor on the primary cilium, thus inducing downstream ERK signaling for MMPs expression. Disruption of primary cilium inhibits trophoblast invasion even in the presence of EG-VEGF, supporting the importance of cilia-mediated EG-VEGF signaling [8]. Besides, fewer primary cilia are observed in the placenta tissues of pregnant women who suffered from preeclampsia, suggesting loss of primary cilia plays an important role in developing preeclampsia. Preeclamptic placenta is a hypoxic microenvironment, and HIF-1α is highly expressed in syncytiotrophoblasts of preeclamptic placenta [118]. Thus, HIF-1α might facilitate the resorption of primary cilia and disrupting the EG-VEGF signaling. Interestingly, this hypothesis is further confirmed by the effect of aspirin on preeclampsia. Aspirin has been used for treating preeclampsia for decades in clinic. Recent studies show that aspirin induces primary cilia and suppresses the expression of soluble fms-like tyrosine kinase 1 (sFLT1), a known marker of preeclampsia, thus promoting trophoblast invasion [81, 141]. A recent study analyzing a cohort of 23, 604 women who had information on placental pathology and aspirin intake during pregnancy revealed that aspirin use reduced risks of having hypoxia-related placental pathology such as thrombus, infarct, fibrin deposition, hydatid, cyst, and calcification [177]. These data provide the potential molecular mechanism by which aspirin prevents preeclampsia maybe via maintaining primary cilia. However, the precise role of HIF-1α in regulating placental hypoxic ciliopathy still needs to be further investigated.

#### Endometriosis

**Fig. 6:**
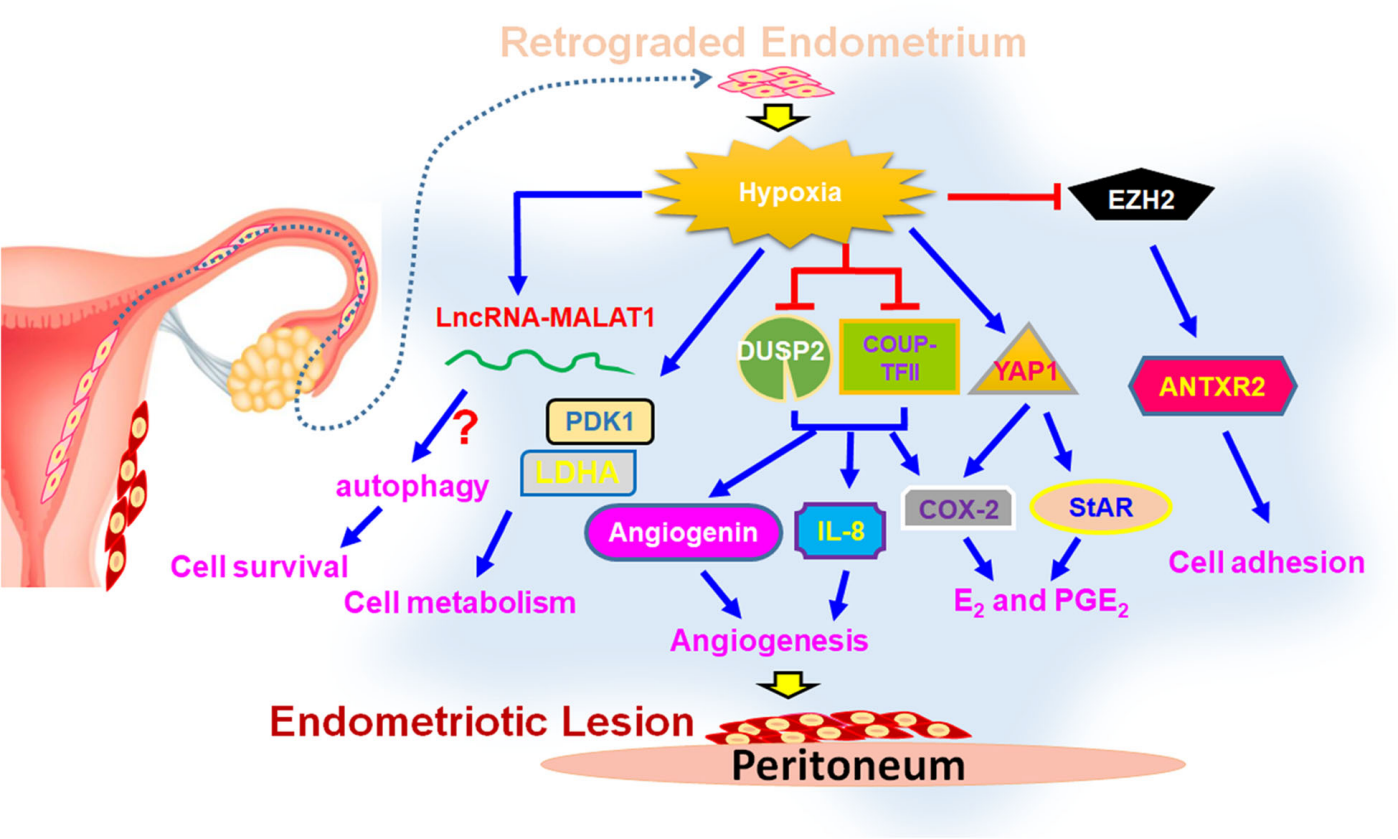
Impacts of hypoxia on endometriosis pathogenesis. Shed-off endometrial tissues will immediately suffer hypoxic stress when it retrogrades into the peritoneal cavity during menstruation. Hypoxia regulates numerous downstream target genes involved in different cellular processes including cell survival, metabolism, angiogenesis, E_2_ and PGE_2_ production, and cell adhesion to help the development of endometriosis

Endometriosis is one of the most common gynecological diseases that reduces life quality and fertility of patients worldwide. It is characterized by the presence of endometrial tissue outside the uterine cavity and the incidence rate of endometriosis is around 8 to 15% in women of the reproductive age [47]. The etiology of endometriosis remains unknown, but a recent report suggests that hypoxia is the driving force of endometriosis [170]. The notion is based on a series of investigations that provided crucial evidence to support the role of hypoxia during the development of endometriosis. Some key findings are summarized below (Fig. 6).

##### Hypoxia and cell adhesion

Endometriosis is initiated by retrograde menstruation. The retrograded endometrial tissues need to land on the surface of organs in the peritoneal cavity and implant in order to survive ectopically. Two challenges that the retrograded cells have to face are hypoxic stress and adhesive ability. Recently, two studies reported that hypoxia enhances cell adhesive ability of the endometrial stromal cells by inducing the expression of cell adhesion molecules such as integrin α5, αV, β3, and β5 via TGF-β1/Smad signaling and anthrax toxin receptor 2 (ANTXR2) via HIF-1α-dependent manner [84, 87]. Furthermore, hypoxia-induced ANTXR2 expression is mediated by downregulation of EZH2 causing epigenetic change in ANTXR2 locus [84]. Treatment with inhibitors of TGF β1 receptor and ANTXR2 significantly attenuates hypoxia-induced cell adhesion in normal endometrial stromal cells [84, 87]. Furthermore, ANTXR2 inhibitor can prevent and reduce endometriotic lesion formation in the mouse model of endometriosis [84], revealing its therapeutic potential for endometriosis. Taken together, it is indicated that hypoxia can promote the development of endometriosis via increasing cell adhesive ability.

##### Hypoxia and hormone production

Previous studies have revealed that both estrogen (E_2_) and prostaglandin E_2_ (PGE_2_) are crucial factors for the development of endometriosis [21, 172] and the enzymes control the rate limiting steps of E_2_ (aromatase and steroidogenic acute regulatory protein, StAR) and PGE_2_ (cyclooxygenase-2, COX-2) biogenesis are aberrantly expressed in endometriotic stromal cells [9, 55, 153, 173]. It was later revealed that hypoxia upregulates both StAR and COX-2 expression. Hypoxia represses dual-specificity phosphatase-2 (DUSP2) expression and contributes to increase COX-2 expression via activation of ERK and p38 signaling pathways [171]. Furthermore, hypoxia also inhibits chicken ovalbumin upstream promoter-transcription factor II (COUP-TFII) to de-repress COX-2 expression in endometriotic stromal cells [85]. Finally, recent study has identified that hypoxia promotes YAP1 activation, which leads to StAR and COX-2 overexpression [83]. The same study also reported that inhibition of YAP1 by its inhibitor, verteporfin, not only decreases E_2_ and PGE_2_ production but also causes the regression of endometriotic lesion in the mouse model of endometriosis [83]. These data indicate that retrograded endometrial tissues can produce E_2_ and PGE_2_ via the assistance of hypoxia to support their growth and development.

##### Hypoxia and angiogenesis

To sustain the growth of endometriotic lesion in the hostile peritoneal cavity, new blood vessels must be established to provide oxygen and nutrient. Heterograft animal model of endometriosis by implanting human eutopic endometrium into severe combined immunodeficiency mice has identified that hypoxia pretreatment for eutopic endometrium significantly increases the level of VEGF to promote cell proliferation and angiogenesis in vivo [93]. Critically, numerous studies have demonstrated the effects of hypoxia on angiogenesis via different mechanisms in endometriosis. Among them, leptin and VEGF-A are two well-known angiogenic factors upregulated by hypoxic treatment in normal endometrial stromal cells [133, 169]. In addition, hypoxia-induced miR-20a causes prolonged activation of ERK singling results in increasing several angiogenic factor including CYR61 and osteopontin [86]. Furthermore, hypoxia can increase angiogenin and IL-8 expression by downregulation of COUP-TFII and DUSP2 expression, respectively [44, 54]. More importantly, IL-8 receptor inhibitor, reparixin, and YAP1 inhibitor, veterporfin, inhibit angiogenesis and the growth of endometriotic lesion in the animal model of endometriosis [54, 83]. Taken together, these findings reveal the critical role of hypoxia-induced angiogenesis during the development of endometriosis.

##### Hypoxia and autophagy

Peritoneal cavity is an unfavorable microenvironment for the retrograded endometrial tissues due to lack of blood vessel to supply oxygen and nutrients. The study has reported that the higher level of oxidative stress-induced DNA damage is observed in endometriotic specimen compared to the normal endometrial tissues [33]. Furthermore, primary endometriotic stromal cells produce more ROS than normal endometrial stromal cells [24]. Therefore, how endometriotic cells survive under this stressful condition is an intriguing question. Previously, autophagy, a cellular mechanism to remove or recycle unnecessary or dysfunctional components to produce energy, has been thought to play crucial roles in protecting cells from oxidative stress-induced cell apoptosis [99, 104]. Indeed, autophagy is reported to be upregulated in the ovarian endometriosis [6, 89] and blocking hypoxia-induced autophagy enhances apoptosis of endometrial stromal cells [88]. Recently, it is further demonstrated that hypoxia-induced long non-coding RNA MALAT1 (lncRNA-MALAT1) is involved in autophagy to protect cells from apoptosis [88]. However, the underlying mechanism that how lncRNA-MALAT1 regulates hypoxia-induced autophagy is still unclear. Besides autophagy, the change of metabolic phenotype in endometriotic lesions may also favor the development of endometriosis. For example, higher levels of glycolysis-related genes such as pyruvate dehydrogenase kinase 1 and lactate dehydrogenase A have been reported in endometriotic specimens and stromal cells, respectively [73, 181]. Furthermore, hypoxia-upregulated pyruvate dehydrogenase kinase 1 has prevented cell death induced by H_2_O_2_ or low nutrient treatment [73]. In summary, hypoxia-induced autophagy and change of cell metabolism may help retrograded endometrial tissues to adapt to the hostile microenvironment and favor the development of endometriosis.

## Conclusion and perspective

The mechanisms of cellular response to hypoxia contribute to stress-induced pathophysiological outcomes. Previous studies established the fundamental concept of hypoxia biology, while recent advances in molecular and cellular biology such as non-coding RNA and microvesicle accelerate us to demonstrate a more complete and complex view of the regulatory network under hypoxia. Notably, most of these new findings were found to be closely integrated in the canonical hypoxia pathway, suggesting a fine-tuned cellular machinery with multiple pathways cooperatively, synergistically, or mutual exclusively work together to modulate the transduction of hypoxia signaling. Identification of these new sensors, messengers, and functional modulators not only advances our knowledge of hypoxia biology but also provides insights into the development of potential diagnostic and therapeutic approaches.

## Data Availability

Not applicable.

## Abbreviations

4E-BP1: EIF4E-binding protein 1
4E-T: EIF4E-transporter
Act1: Activator 1
AGPAT2: Acylglycerol-3-phosphate acyltransferase 2
AKI: Acute kidney injury
AMPK: AMP-activated protein kinase
ANG: Angiopoietin
ANTX2: Anthrax toxin receptor 2
ARNT: Aryl hydrocarbon nuclear translocator
ATF6: Activating transcription factor 6
ATGL: Adipose triglyceride lipase
Bcl-2: B-cell lymphoma 2
Bad: Bcl-2 associated agonist of cell death
Bax: Bcl-2-associated X protein
Bcl-xL: B-cell lymphoma-extra large
BDNF: Brain derived neurotrophic factor
Bid: BH3 interacting domain death agonist
BNIP3: Bcl-2 interacting protein 3
C/EBP: CCAAT/enhancer binding protein
CA: Carbonic anhydrase
CASP3: Caspase-3
CBP: CREB-binding protein
CD36: CD36 molecule
CKD: Chronic kidney disease
COUP-TFII: Chicken ovalbumin upstream promoter-transcription factor II
COX-2: Cyclooxygenase-2
CPT1A: Carnitine palmitoyltransferase 1A
CRD: Apoptosis repressor with caspase recruitment domain
CSC: Cancer stem cell
CYR61: Cysteine rich angiogenic inducer 61
DAPK1: Death-associated protein kinase 1
DEC1: Differentiated embryo-chondrocyte expressed gene 1
DUSP2: Dual-specificity phosphatase-2
E_2_: Estrogen
ECM: Extracellular matrix
EFNA3: Ephrin A3
EGFR: Epidermal growth factor receptor
EG-VEGF: Endocrine gland-derived vascular endothelial growth factor
EIF4E: Eukaryotic translation initiation factor 4E
EMT: Epithelial-to-mesenchymal transition
ER: Endoplasmic reticulum
ERK: Extracellular signal-regulated kinase
ESRD: End-stage renal disease
EZH2: Enhancer of zeste 2 polycomb repressive complex 2 subunit
FABP: Fatty acid binding protein
FAS: Fatty acid synthase
FATP1: Fatty acid transport protein 1
FGF: Fibroblast growth factor
FIH: Factor inhibiting HIF
FOXO1: Forkhead box protein O1
GLUT: Glucose transporter
H3K4Ac: Histone 3 lysine 4
HDAC3: Histone deacetylase 3
HIF: Hypoxia inducible factor
HK: Hexokinase
HRE: Hypoxia responsive element
IDH: Isocitrate dehydrogenase
IKK: Inhibitor of κB kinase
IL-8: Interleukin 8
ILK: Integrin-linked kinase
IRE1: Inositol-requiring protein 1
IκB: Inhibitor of NF-κB
KDM: Histone lysine demethylase
LDHA: Lactate dehydrogenase A
lncRNA-MALAT1: Long non-coding RNA MALAT1
LOX: Lysyl oxidase
MAP K6: Mitogen-activated protein kinase 6
MMP: Matrix metalloproteinases
MO25: Mouse protein 25
mTOR: Mammalian target of rapamycin
NF-κB: Nuclear factor-κB
NOS3: Nitric oxide synthase 3
PAI-1: Plasminogen activator inhibitor-1
PARP: Poly (ADP-ribose) polymerase
PDCD4: Programmed cell death 4
PDGF-B: Platelet-derived growth factor B
PDK: Pyruvate dehydrogenase kinase
PERK: PKR-like ER kinase
PGC-1α: PPARγ coactivator 1-α
PGE2: Prostaglandin E2
PGK-1: Phosphoglycerate kinase 1
PHDs: Prolyl-hydroxylases
PPARγ: Peroxisome proliferator-activated receptor γ
PTP1B: Protein tyrosine phosphatase 1B
REDD1: Regulated in development and DNA damage responses 1
RHEB: Ras homolog enriched in brain
ROS: Reactive oxygen species
sFLT1: Soluble Fms-Like tyrosine kinase 1
SIRT1: Sirtuin 1
SNAI1: Snail family transcriptional repressor 1
Spry1: Sprouty 1
SREBP1: Sterol regulatory element-binding protein 1
StAR: Steroidogenic acute regulatory protein
TAC: Transaortic constriction
TGF-β: Transforming growth factor beta
TKRs: Tyrosine kinase receptors
TNF-α: Tumor necrosis factor-α
TWIST1: Twist family bHLH transcription factor 1
UCP1: Uncoupling protein 1
UPR: Unfolded protein response
VEGF: Vascular endothelial growth factor
VEGF-A: Vascular endothelial growth factor A
VHL: E3 ubiquitin ligase von Hippel–Lindau
WDR5: WD repeat domain 5
XBP1: X-box binding protein 1
XIAP: X-linked inhibitor of apoptosis protein
YAP1: Yes Associated Protein 1
ZEB1: Zinc finger E-box-binding homeobox 1
